# Safe Surgery During the COVID-19 Pandemic

**DOI:** 10.1007/s13679-021-00458-6

**Published:** 2021-10-28

**Authors:** Rishi Singhal, Luke Dickerson, Nasser Sakran, Sjaak Pouwels, Sonja Chiappetta, Sylvia Weiner, Sanjay Purkayastha, Brij Madhok, Kamal Mahawar

**Affiliations:** 1 Upper GI Unit, University Hospitals Birmingham NHS Foundation Trust, Birmingham Heartlands Hospital, Birmingham, UK; 2 Department of General Surgery, Leighton Hospital, Crewe, UK; 3 Director Bariatric Centre, Department of Surgery, Emek Medical Centre, Afula, Israel; 4 The Rappaport Faculty of Medicine, Technion, Haifa, Israel; 5 Department of Intensive Care Medicine, Elisabeth-Tweesteden Hospital, Tilburg, the Netherlands; 6 Head Obesity and Metabolic Surgery, Ospedale Evangelico Betania, Naples, Italy; 7 Department of Obesity and Metabolic Surgery, Krankenhaus Nordwest, Frankfurt am Main, Germany; 8 Imperial College London, London, UK; 9 Royal Derby Hospital, Derby, UK; 10 Bariatric Unit, South Tyneside and Sunderland NHS Trust, Sunderland, UK

**Keywords:** COVID-19, Surgery, Surgical outcomes, Pre-operative screening, Testing, Vaccination

## Abstract

***Purpose of Review*:**

Coronavirus Disease-2019 (COVID-19) has had an enormous impact on all aspects of healthcare, but its effect on patients needing surgery and surgeons has been disproportionate. In this review, we aim to understand the impact of the pandemic on surgical patients and teams. We compiled the emerging data on pre-operative screening methods, vaccinations, safe-surgery pathways and surgical techniques and make recommendations for evidence-based safe-surgical pathways. We also present surgical outcomes for emergency, oncological and benign surgery in the context of the pandemic. Finally, we attempt to address the impact of the pandemic on patients, staff and surgical training and provide perspectives for the future.

***Recent Findings*:**

Surgical teams have developed consensus guidelines and established research priorities and safety precautions for surgery during the COVID-19 pandemic. Evidence supports that surgery in patients with a peri-operative SARS-CoV-2 infection carries substantial risks, but risk mitigation strategies are effective at reducing harm to staff and patients.

***Summary*:**

Surgery has increased risk for patients and staff, but this can be mitigated effectively, especially for elective surgery. Elective surgery can be safely performed during the COVID-19 pandemic employing the strategies discussed in this review.

## Can Surgery Be Done Safely During the COVID-19 Pandemic?

## Introduction

Coronavirus Disease-2019 (COVID-19) has had an enormous impact on surgical patients. In this review, we aim to understand the impact of the pandemic on surgery in its totality. We will review safety precautions adopted by surgical teams and outcomes of different types of surgery performed with the full knowledge of the pandemic. We will also examine the steps needed to mitigate the effects of the pandemic on resources and training.

## Background

In December 2019, a respiratory disease was identified in the Wuhan province of China, later identified as a novel virus strain. Severe Acute Respiratory Syndrome Coronavirus-2 (SARS-CoV-2) was responsible for the Coronavirus Disease 2019 (COVID-19) that then spread rapidly across the globe, posing a danger that the world had not seen since the second world war [1, 2]. Despite some countries managing to control case numbers, many continue to struggle, and the number of newly diagnosed patients is still rising in many parts of the world. The success of multiple viable vaccines threatens to be undone by the rise of mutated variants of SARS-CoV-2 which pose new challenges in multiple countries [3].

Surgeons entered this pandemic with the knowledge that perioperative SARS-CoV-2 infection was associated with high morbidity and mortality. One large study estimated the 30-day mortality to be 23.8%, with worse outcomes in those undergoing emergency surgery (25.6% vs. 18.9%) [4•]. This and other such studies prompted the cancellation of millions of surgical procedures worldwide [5]. The COVIDSurg Collaborative estimated that over 28 million surgical procedures in 190 countries would be cancelled in the estimated 12-week ‘pandemic peaks’ for each country [6]. Cancer surgery cancellation rates were estimated at 37.6%. For benign surgery, cancellation rates were estimated to be much higher at 81.6% and these comprised 90.2% of all cancellations [6].

Furthermore, there were concerns at the beginning of the pandemic regarding the nosocomial transmission of the virus and the risk to operating theatre and endoscopy staff. These concerns were not based on scientific evidence but understandable in the context of the high-profile deaths of several surgeons early in the pandemic [7].

Regarding laparoscopic surgery, the aerosolisation of the virus appeared to be the main concern based on evidence with other viruses, and the use of diathermy was questioned even with open procedures [8, 9]. The presence of coronavirus within faeculent material further heightened anxieties regarding GI procedures and endoscopy [10]. The combination of this led to the initial recommendation by many respectable organisations that laparoscopic surgery should be avoided during the pandemic.

## Consensus Statements and Safety Precautions

All these factors meant that there was an urgent need to inform surgical practice during the COVID-19 pandemic and research priorities were established [11]. The evidence vacuum for surgery during the pandemic further led to urgent development of consensus statements to inform practice and allow for the safe resumption of surgery [12–17]. Over the last year or so, surgeons from around the world have attempted to examine these recommendations, adapting our response to this novel threat. In this review, we summarise the current evidence base informing surgical practice during the COVID-19 pandemic.

### Patient Risk Mitigation

Though the initial response of the surgical community was to cancel the surgical activity, we are recognising that the pandemic may not dissipate anytime soon, and postponing all non-urgent procedures indefinitely is no longer viable. Identifying factors associated with worst outcomes and developing protective strategies is crucial for safe resumption of elective surgery. Several strategies have been examined to reduce the risk of perioperative SARS-CoV-2 infection which is associated with high morbidity and mortality. In the following paragraphs, we examine the most important amongst these.

### Pre-operative Screening and Testing

All the varied presentations of COVID-19 were not fully appreciated at the beginning of the pandemic. For example, anosmia was only added to the UK’s National Health Service (NHS) list of symptoms on the 18th of May 2020 [18–20]. An improved understanding of symptoms has enabled better diagnosis of COVID-19, but up to 20% of infected persons are asymptomatic throughout their infection [21]. Not only that, a mean incubation period of 5.1 days (95% CI 4.5–5.8) means many patients may well be incubating when they attend for surgery [22]. Asymptomatic and incubating patients will not be picked up by symptomatic screening alone.

The prevalence of asymptomatic infection necessitates universal pre-operative screening. Nasopharyngeal swabs for detecting COVID-19 RNA via PCR (RT-PCR) remain the mainstay of initial screening for COVID infection given its ease of use and high sensitivity of nearly 75.0% [23•]. The major drawback of PCR testing is the time required for results which is at least an hour. This is especially pertinent in centres where PCR testing is not performed ‘in house’.

Lateral flow testing is point of care testing for COVID-19 antigens, but this is limited with pooled sensitivities from 18 studies of 1857 patients of 66% (49.3 to 79%) with higher sensitivities in non-commercial test kits and with hospitalised patients [24]. Loop-mediated isothermal amplification (LAMP) testing is an emerging testing method that negates the time delay and cost of PCR testing whilst retaining the accuracy (sensitivity of 75% and 95% and specificity of 99–100% depending on whether RNA extraction was performed) [25]. Combining RT-PCR with CT thorax for excluding pre-operative COVID-19 infection does not improve the yield significantly and is resource intensive [26, 27].

Serological testing for anti-SARS-CoV-2 immunoglobulin detection of previous COVID-19 exposure is currently being investigated for its role in predicting treatment outcomes off the back of the RECOVERY trial [28]. The trial showed that seronegative patients were more likely to succumb to COVID-19, and receive mechanical ventilation and less likely to be discharged than seropositive patients. Administration of casirivimab and imdevimab reduced 28-day mortality in seronegative patients (rate ratio 0·80; 95% CI 0·70–0·91; *p* = 0.0010) potentially showing a role of individualised treatment. Pilot studies are being carried out in parts of England to investigate this further. The role of serology in the acute situation is limited by high false-negative rates in the first week after exposure (44–87%) [24]. Diagnostic accuracy increases with time from exposure with 100% of those infected having anti-SARS-CoV-2 IgG 12 weeks after infection [29, 30]. Early in the pandemic, some institutions performed serological testing as there was concern regarding the false-negative rates of viral RNA detection using PCR testing. Serological testing to assess exposure of healthcare workers has been performed early in the pandemic, but its use has largely become obsolete with increased availability of PCR, lateral flow, and LAMP testing.

Regarding the timings of the swabs, the current consensus seems to be for a swab in the 72 h leading up to surgery with the surgery being conditional on a negative result or clinical indication to justify the risk if it is not possible to wait for a negative test (such as emergency surgery) [16, 31]. For screening of symptoms, detailed questionnaires have been proposed [18]. Generally, it would seem reasonable to postpone surgery if the patient or any of the immediate family members have had a fever of 37.5 °C or more, flu-like symptoms (nasal discharge, sore throat, cough), loss of taste or smell, diarrhoea, headache, body ache, tiredness, or shortness of breath in the 2 weeks leading up to surgery [32, 33]. The risk of mortality due to COVID-19 in an asymptomatic patient with a negative RT-PCR for SARS-CoV-2 has been estimated to be very low [34].

#### Management Patients with Positive Tests

If clinically possible, the operation should be deferred in case of a positive RT-PCR test, or if the patient has symptoms suggestive of COVID-19 [16, 31]. There is higher adjusted mortality rates in those with pre-operative COVID-19 diagnosis at different time points with significant differences as 0–2 weeks, 3–4 weeks and 5–6 weeks pre-operatively (OR 3.22 (2.55–4.07), OR 3.03 (2.03–4.52) and 2.78 (1.64–4.71) respectively, all *p* ≤ 0.001). [35]. There is no difference after 7 weeks (OR 1.02 (0.66–1.56)). These risks are relatively consistent across major and minor surgeries with minor surgery carrying less risk overall (OR 2.37 (2.11–2.67) and at each of the time points (0–2 weeks = OR 2.36 vs. 4.92, 3–4 weeks = OR 2.23 vs. 4.68 5–6 weeks = OR 2.06 vs. 4.35 and > 7 weeks = OR 0.81 vs. 1.81) [35]. Those with persisting symptoms at 7 weeks are at higher risk (OR 5.96 (3.24–8.68) than asymptomatic (OR 1.3 (0.59–2.01)) and resolved 2.43 (1.42–3.44). In the paediatric cohort, there is limited data but a minimum 28-day delay has been suggested for asymptomatic patients positive for SARS-CoV-2 in a paediatric cohort [36]. In patients undergoing cancer surgery, a 3-week wait has been recommended [37]. The waiting time for patients who are symptomatic for COVID-19 (with or without a positive test) should naturally be guided by the clinical course of the disease and the indication of surgery.

Recommendations: Elective patients should have PCR testing 72 h prior to surgery. Positive tests should result in operative delay of at least 3 weeks in time-critical surgery or 7 weeks in non-time critical surgery. Those who remain symptomatic should have individualised decisions made by the MDT including surgeons and anaesthetists. Emergency surgical patients should be triaged with rapid testing (lateral flow) whilst concurrent PCR testing is performed to validate these results. Serological testing may come into routine pre-operative workup to guide management should the patient develop COVID-19 but is not recommended as standard at present.

### Preoperative Self-isolation

Preoperative self-isolation can reduce the odds of inadvertent surgery on asymptomatic or incubating patients and is relevant to non-time critical surgery. In a study on an elective orthopaedic cohort from Japan, the authors found that self-isolation for 2 weeks before surgery was highly effective and none of the 304 patients who completed the programme successfully later tested positive for the virus on the RT-PCR test [32]. Other institutions have recommended 72 h of isolation.

Pre-operative self-isolation does have implications on patients including effects on vocation, for those not able to work from home and social isolation. Despite this, in a retrospective analysis of elective upper limb/hand operations at a single US institution, the authors found that majority of patients agreed or strongly agreed that COVID testing (88.1%, 310/352) and quarantining for 72 h pre-operatively (66.7% 245/352) is necessary [38]. Only 53/352 felt they should not have to adjust their behaviour after a negative test.

At the same time, recently published GENEVA study of 7704 bariatric surgery patients from 42 patients observed that preoperative self-isolation did not reduce the incidence of postoperative symptomatic COVID-19 [39].

Recommendation: Preoperative self-isolation is a sensible, low-cost, easy-to-implement precaution that is generally well-understood by patients, but scientific evidence to recommend routine preoperative self-isolation for 2 weeks for all surgical patients is lacking and obviously such a strategy is not without its own social and financial implications for patients. We do not hence recommend routine preoperative self-isolation for 2 weeks for all surgical patients.

We would however recommend preoperative self-isolation for 2 weeks for all high-risk major elective surgery (for example major cancer resections) where the implications of perioperative SARS-CoV-2 infection are significantly greater. For all other surgeries, 72 h of isolation after a negative test may be more practical.

### ‘COVID-19 Free or Minimal’ Pathways

There is evidence to suggest that ‘COVID-19 free’ or ‘COVID-19 minimal’ surgical pathways reduce the risk of postoperative pulmonary complications compared to no defined pathway [40]. This generally means a separate hospital (or a separate ward and theatres if that is not possible) for all elective surgery patients. It is unclear if this also means separate healthcare staff and regular testing for them. Current guidelines from the Joint Royal Colleges of Surgeons in the UK advocate staff be tested twice a week, and many trusts in the UK are recommending either lateral flow or LAMP testing for operating theatre personnel.

The use of such pathways does not lead to the elimination of morbidity and mortality due to COVID-19, but data suggest that the mortality due to COVID-19 is lower when these pathways are used [41–44]. Such COVID-19-free surgical pathways are effective in reducing SARS-CoV-2 infection and overall mortality in patients undergoing cancer surgery suggesting success in reducing nosocomial transmission [45•].

The exact definition of these pathways and their impact on COVID-19 mortality reduction over and above the use of other safety precautions however remain to be examined.

**Fig. 1:**
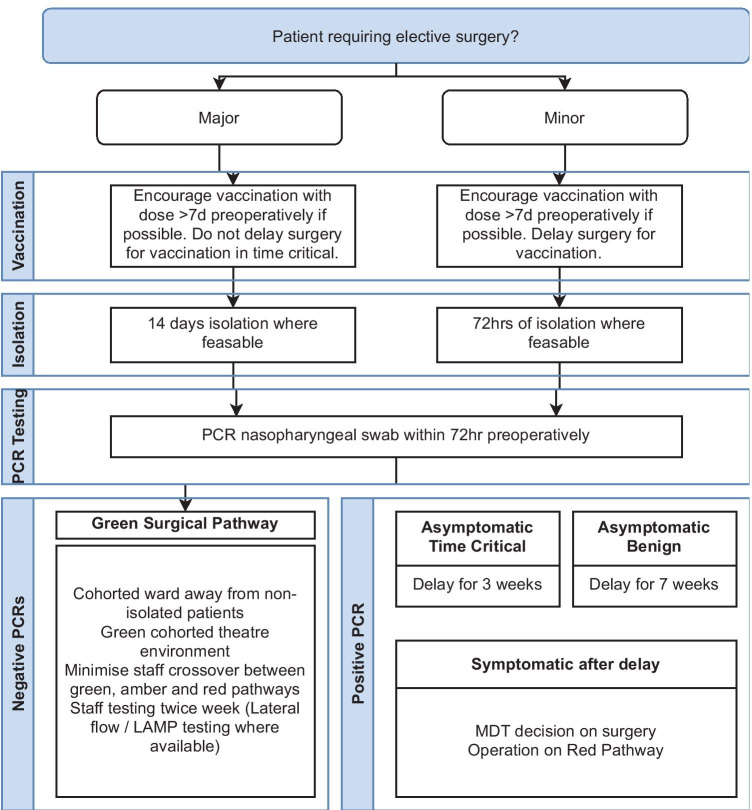
Elective surgical pathway

**Fig. 2:**
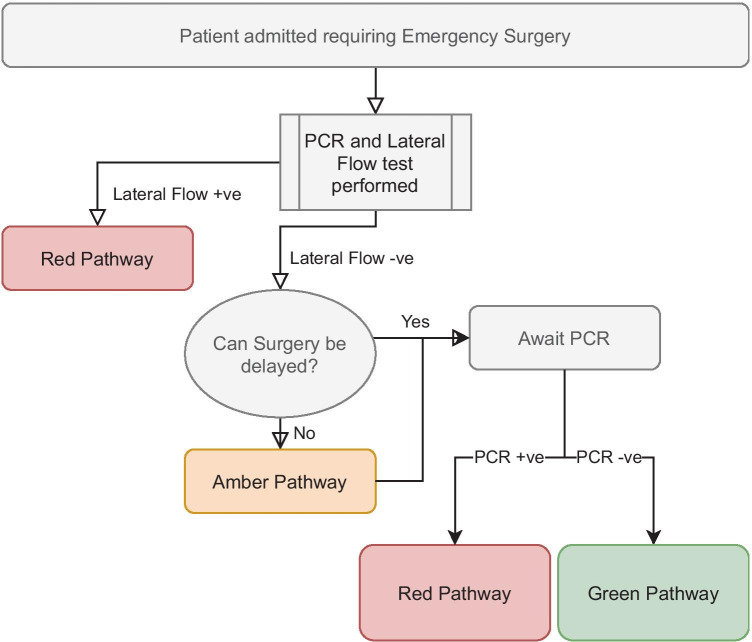
Emergency surgery flowchart

Recommendation: COVID-free pathways are a practical mechanism for reducing inter-patient spread of SARS-CoV-2 and should be implemented where feasible. Figure 1 (Elective) and Fig. 2 (Emergency) depict our suggested recommendations for such pathways.

### Vaccination

There are now several vaccines against this virus that have been approved or are in phase III trials with reported efficacy of ~ 60 to 95% at preventing infection and > 90% for preventing severe disease [46–51]. The efficacy of the COVID-19 vaccinations at reducing disease severity has been proposed as a risk amelioration strategy before elective surgery [52]. Predicted numbers needed to be vaccinated to prevent one COVID-related death/annum varies according to patient age, operative type, and COVID prevalence. The benefit was highest in > 70-year-old patients undergoing cancer operations in high-prevalence areas (NNV = 221), but the benefit of vaccination was seen across all age categories and surgical types. With the rise of variants there is concern about the efficacy of vaccination but early studies have shown good effect at preventing severe infection across the spectrum. For example in Qatar, effectiveness of the Pfizer vaccination against any infection of B.1.1.7 and B.1.351 where the prevalence of these variants are high (> 97%) was 89.5% and 75% respectively and against severe disease 97.4% [53].

Despite this data, there is no clear guidance on vaccination as a protective strategy for patients undergoing elective surgery. Lack of easy access to vaccines in some parts of the world may further make this difficult unless governments include surgical patients into their priority vaccination cohorts. There is some guidance to suggest that surgery should not occur within 7 days of vaccination to allow the immune system to adapt to the effect of vaccination prior to surgical stimulus, but there are no outcomes data on vaccination and surgical morbidity and mortality [54].

With regards to the vaccination attempts, there is huge variation in government vaccination programmes worldwide [55]. Percentages of population vaccinated varies dramatically from 69.81% of Canadians having had at least one dose to < 1% in some African countries. There is disparity across continents with the average African percentage of the population vaccinated at 2.95% compared to high rates in the EU countries (54.87%), Europe (44.96%) and USA (55.27%). South America and Asia have lower rates of population vaccination but larger variation (Singapore 69.27% vs. Vietnam 3.97%). The implications of this on outcomes of global surgical practice are yet to be established. In UK, there have recently been substantial increases in numbers of cases but reduced hospital admissions and mortality relative to the first and second waves. This highlights the role of vaccination in reducing the severity of infection and should hopefully reduce demand on hospital services to allow non-COVID-related work to continue unabated.

Recommendations: Vaccination reduces incidence and severity of SARS-CoV-2 infection including its current variants. It should be recommended for all patients undergoing elective surgery that can wait. Surgical patients should be identified as a priority cohort for vaccination in resource constrained settings where the availability of vaccines is low.

### Risk Factors for Perioperative SARS-CoV-2 Infection

Studies have shown that that 30-day mortality of perioperative SARS-CoV-2 infection is higher with emergency surgery, cancer surgery, prior transplant, immunosuppressed, presentation within the first week after surgery, those > 75 years of age, and ASA > 2 [4•, 56–58]. Such analyses of risk factors for serious morbidity or mortality associated with perioperative SARS-CoV-2 infection will further help us make surgery safer during the pandemic and allow for more focussed use of safety precautions.

When it comes to infection itself, emergency surgery is associated with a greater risk of perioperative SARS-CoV-2 infection (*p* ≤ 0.001), and this is supported in the subgroup analysis of colorectal (*p* ≤ 0.001) and upper gastro-intestinal/hepato-pancreato-biliary surgery (*p* = 0.008) [59].

Laparoscopic surgery seems to be associated with a reduced risk of COVID-19 infection compared to open surgery for major and major complex surgery (*p* = 0.040) [59]. There is further evidence that smoke compositions in laparoscopy and laparotomy are similar but laparoscopy allows for more controlled release [8]. All these factors seem to suggest that laparoscopic surgery is safe during the pandemic.

Recommendations: Emergency surgery is associated with higher risk of perioperative SARS-CoV-2 infection and is also associated with a higher morbidity and mortality from it. Laparoscopic surgery is safe during the pandemic.

#### Postoperative Self-isolation

Evidence is limited on post-operative self-isolation, but this is advocated in the UK intercollegiate guidance where possible [31]. It may be more useful where returns to surgery may be required such as pending histology in breast-wide local excisions. In our view, this should be selectively recommended for at least 2 weeks for the highest risk cohort identified above.

#### Other Safety Measures

Amongst other suggestions that could help reduce perioperative SARS-CoV-2 infection are reduction of the time spent by the patient in the healthcare setting; where possible virtual pre-operative consultations, assessments and postoperative consultation; and use of appropriate personal protective equipment by staff. There is currently little evidence that these would reduce perioperative SARS-CoV-2 infection or mortality but they seem sensible and may have other benefits.

#### Consent for Surgery

The risk and consequences of perioperative COVID-19 vary depending on the nature of the surgery, patient characteristics and pandemic burden within the community. Patients should be made aware of the best possible estimates of COVID-19-specific morbidity and mortality before the surgery. This should form a standard part of all consent during the pandemic. There have been attempts to develop a comprehensive informed consent for use during the pandemic including information on unexpected absence or replacement of staff, risk of perioperative SARS-CoV-2 patients and other unexpected occurrences that cannot be discussed in advance [37].

### Theatre Staff Risk Mitigation

It is well established that frontline healthcare staff are at higher risk than the general public during the COVID-19 pandemic [60]. Healthcare workers were more likely to have COVID symptoms (20.2% vs. 14.4%) and had a higher 30-day risk of a positive test (3.96% vs. 0.33%, multivariate-adjusted HR 11.61).

Adequate personal protective equipment (PPE) is well established as the mainstay of prevention of nosocomial transmission and remains integral to respiratory pathogen disaster plans [61–63]. Personal protective equipment varies according to the degree of protection needed (from basic surgical masks to N95 masks that filter 95% of airborne particles. Adequate protection in the form of N95 masks was not always available at the beginning of the pandemic, which correlated with an increased risk of infection [60, 64]. Appropriate PPE provision should be the highest priority in ensuring staff safety. Other strategies that could help protect staff are vaccination, social distancing, hand hygiene and measures to minimise exposure to aerosols [65]. It is recommended by societies advocating laparoscopic surgery that surgeons employ techniques that reduce smoke production (e.g. lowering energy device settings) and sudden release of pneumoperitoneum. This has additional health benefits for the surgical teams reducing their exposure to the toxic chemicals including carcinogens within the surgical smoke plume [66].

The COVID-19 pandemic has had a significant impact on the mental health and overall well-being of the frontline healthcare staff [67]. In addition, theatre staff were redeployed to medical wards and critical care units, and hence may have become deskilled. Hospital managers need to consider this in planning theatre lists especially if staff are once again re-deployed. We would suggest fewer operations per list than was the norm before the pandemic to facilitate patient safety and adherence to PPE/social distancing guidance.

Recommendations: Staff are at higher risk than the general population from COVID-19 and appropriate provision of FFP 3/N95 masks should be ensured for those dealing with positive patients (in emergency surgery) and basic PPE for those with negative swabs. Staff with underlying health conditions may wish to wear higher levels of protection and such requests should be accommodated.

### Surgical Outcomes

#### Outcomes in Patients with Perioperative SARS-CoV-2 Infection Undergoing Emergency Surgery

We know from several datasets that patients undergoing emergency surgery with perioperative SARS-CoV-2 infection experience significant morbidity and mortality [4, 68]. One of the most important international collaborations assessing the effects of COVID-19 in the perioperative period across all surgical disciplines is the COVIDSurg Collaborative [4]. They showed in an international multicentre collaborative of 235 hospitals in 24 countries that a perioperative SARS-CoV-2 infection was associated with significant morbidity and mortality. They analysed 1128 patients of whom 835 (74.0%) had emergency surgery and 280 (24.8%) had elective surgery between January and March 2020. The 30-day mortality was 23.8% (268 of 1128). In total, pulmonary complications occurred in 577 (51.2%) of the 1128 patients. These findings have also been confirmed by others. For example, De et al. found a mortality of 41.2% in SARS-CoV-2-positive patients undergoing surgery for hip fracture [69]. Similarly, Knisely et al. observed a mortality of 16.7% in SARS-CoV-2-positive patients undergoing urgent or emergency surgery compared to 1.4% in those without [70].

#### Outcomes in Patients Undergoing Cancer Surgery During the COVID-19 Pandemic

Patients undergoing any cancer surgery form a particularly interesting cohort of semi-urgent surgical patients whose surgery cannot be postponed indefinitely but at the same time can be deferred for a short duration in patients with SARS-CoV-2 infection. Generally, the outcomes of cancer surgery performed with full awareness of the pandemic have been satisfactory with morbidity and mortality comparable to pre-pandemic data and very low morbidity/mortality attributable to COVID-19. We list salient findings of some of the important studies on this topic in the following paragraphs.

In a large study reporting on the outcomes of elective colorectal cancer surgery during the pandemic, authors found that postoperative SARS-CoV-2 infection occurred in 3.8% (78 of 2073) patients and was independently associated with mortality (odds ratio (OR): 16.90, 95 CI: 7.86–36.38) [71]. Compared with pre-pandemic data, authors observed a shorter length of stay (6 vs. 7 days) but higher mortality (1.7% vs. 1.1%) [71]. There were fewer anastomotic leaks (4.9% vs. 7.7%). This reduction in leaks was offset by a marginal increase in stoma formation (34.2% vs. 27.2%) [71].

In another similar study, Xu et al. found that out of 710 patients with colorectal cancer who underwent curative resection during the pandemic [72], surgeries were performed laparoscopically in 49.4%, significantly higher than the 39.5% during the same period in 2019. The proportion of major complications during the pandemic was not significantly different from that of the control group. The mean hospital stay was significantly longer than that of the control group. They concluded that colorectal cancer patients confirmed to be infection-free can receive routine treatment.

Glasbey et al. studied 9171 patients from 447 hospitals in 55 countries [45•]. Of these, 2481 were operated on in COVID-19-free surgical pathways [45•]. Patients who underwent surgery within COVID-19-free surgical pathways were younger with fewer comorbidities than those in hospitals with no defined pathway but had similar proportions of major surgery. After adjustment, pulmonary complication rates were lower with COVID-19-free surgical pathways (2.2% vs. 4.9%; adjusted odds ratio [aOR], 0.62; 95% CI, 0.44 to 0.86). This was consistent in sensitivity analyses for low-risk patients (American Society of Anaesthesiologists grade 1/2), propensity score-matched models, and patients with negative SARS-CoV-2 preoperative tests. The postoperative SARS-CoV-2 infection rate was also lower in COVID-19-free surgical pathways (2.1% vs. 3.6%; aOR, 0.53; 95% CI, 0.36 to 0.76). The authors concluded that within available resources, dedicated COVID-19-free surgical pathways should be established to provide safe elective cancer surgery during current and before future SARS-CoV-2 outbreaks.

In another study of head and neck cancer, treated during the COVID-19 pandemic, 1137 patients were analysed [73]. Most of these patients had an oral cavity (38%) or thyroid (21%) cancers. The overall 30-day mortality was 1.2%. A total of 29 (3%) patients tested positive for SARS-CoV-2 within 30 days of surgery. Thirteen of these patients (44.8%) developed severe respiratory complications, and 10.3% of these patients died. Twenty-two percent were operated on in cold centres.

In a multicentre study in four European oesophageal cancer referral centres, authors did not find COVID-19 pre- or postoperatively in any of their 139 patients who underwent surgery during the pandemic [74]. There was further no difference in the rate of respiratory failure requiring mechanical ventilation (13.7% vs. 8.3%, *p* = 0.127) and the number of pulmonary complications (32.4% vs. 29.9%, *p* = 0.646) between the pandemic cohort and the control cohort. Overall, postoperative morbidity and mortality rates were also comparable between both cohorts. History and reverse transcription polymerase chain reaction (RT-PCR) were used as preoperative screening methods to detect a possible severe acute respiratory syndrome coronavirus 2 (SARS-CoV-2) infection in all centres.

Others have shown similar data in patients undergoing surgery for endocrine cancers and urology cancers [75, 76]. Based on these studies, we believe that cancer surgery should continue during the pandemic with locally appropriate safety precautions that should probably include screening for symptoms and close contact with COVID-19 patients in 2 weeks leading up to surgery, preoperative RT-PCR testing, preoperative self-isolation after the testing (and for 2 weeks in those deemed at high risk of complications from perioperative SARS-CoV-2 infection), treatment in COVID-free or COVID-minimal facilities, and postoperative self-isolation for 2 weeks in the high-risk group.

#### Outcomes in Patients Undergoing Elective Benign Surgery During the COVID-19 Pandemic

The impact of the pandemic on elective benign surgery is likely to be different from that on emergency or cancer surgery as this type of surgery can be postponed for longer durations and allows for all appropriate safety precautions to be used. This is probably the reason that data on elective surgery during the pandemic show very low morbidity and mortality due to COVID-19 and outcomes generally similar to pre-pandemic data.

The ‘Global 30-day outcomes after bariatric surgery during the COVID-19 pandemic’ (GENEVA) showed that bariatric and metabolic surgery could be performed safely during the pandemic [39]. The complete dataset from this study has been reported recently for adults and adolescents [77, 78]. In this study, 499 surgeons from 185 centres in 42 countries provided complete data on 7704 patients. Forty-three patients (0.56%) developed symptomatic COVID-19 post-operatively with a higher risk in non-whites. The risk of postoperative COVID-19 risk was greater if surgery was performed during a local peak. The authors concluded that bariatric and metabolic surgery could be safely performed during the COVID-19 pandemic.

Similarly, others have shown outcomes in other cohorts of benign surgery patients that seem similar to morbidity and mortality before the pandemic and very low morbidity and mortality attributable to COVID-19 [79–81]. Hence, we believe elective benign surgery should continue during the pandemic with precautions similar to those we have suggested above for cancer surgery. However, the nature of this type of surgery does allow more time for vaccination and postponement for short periods if deemed necessary for those at high risk.

#### Prioritisation and Resource Allocation

Inevitably, there is going to be stiff competition for limited resources during and after the COVID-19 pandemic. Surgical teams will necessarily have to prioritise patient groups and conditions that will need to be treated first. Guidelines have also been issued by surgical bodies to facilitate this work [82].

Kursumovic et al. retrospectively looked at the impact of COVID on anaesthetic services during the second wave of the COVID pandemic in the UK (01 November 2020 to 1 February 2021) [83]. Theatre closure reached highs of 42%, and in those centres maintaining theatre work, the capacity decreased substantially (near normal = 48 to 32% and < 50% productivity = 10 to 27%). Those theatres that were open were drastically understaffed owing to redeployment or absence [83].

One cannot argue against emergency surgery and cancer surgery being given the top priority in these circumstances, but choosing amongst the vast range of benign elective surgeries can be a challenging task. These decisions will inevitably depend on local priorities and resources, but hospital managers and surgeons should be careful to not discriminate against any group of patients. Patients suffering from obesity, older patients and high-risk patients could fall into this category.

#### Impact on Surgical Trainees and Training

The development of surgical skills is a long and arduous process. The COVID-19 pandemic has interfered with this process in many ways. It has reduced the exposure of the trainees to surgery as in many places only essential members of the staff are admitted to the operating room to protect patients and health care providers [84]. Secondly, cancellation of millions of elective and semi-urgent procedures during the pandemic has reduced surgical volume and exposure around the world [6, 85, 86]. Thirdly, increased usage of telemedicine and virtual clinics has reduced opportunities for patient interaction and examination [85, 87]. Moreover, some surgical trainees have taken time off work and many were redeployed to COVID departments [88–90]. This has particularly affected trainees in their final year of training [91].

There have also been some positives with novel avenues for residents and fellows who have had more time and opportunity for performing academic work, balancing family commitments, and rotas during the pandemic [85]. There is also some evidence to suggest that reduced surgical volume has enabled trainers to train trainees [71].

Hoopes et al. reviewed the literature regarding gynaecological surgical simulation and identified nine categories of resources in preventing skill decay [92]. Surgical simulation can help develop the psychomotor, visual-spatial and cognitive skills — all important for surgical performance. They should be considered in the design of a remote surgical training curriculum.

Schlegl et al. established a reproducible distance education curriculum for teaching students basic surgical skills using homemade tools, like shoes, fishing lines, kitchen sponge as suture pads and cardboard box and webcam as a pelvitrainer [93]. Oakland et al. proposed ‘surgical simulation kits’, which include all instruments necessary to simulate a procedure at home. In this case, each ‘auricular hematoma surgical kit’ was linked to an online module and the procedure was discussed virtually with a senior surgeon [94].

We believe the use of simulation and regular recording of videos of procedures and detailed feedback on the technical aspects can offset some of the loss of surgical training seen during the pandemic. However, trainers will need to be provided additional time for such feedback, and training programmes should consider developing dedicated surgical mentors specifically for the development of technical surgical skills.

#### Looking to the Future

COVID-19 has taught us many harsh lessons about our preparedness for this pandemic that whilst predicted was not adequately prepared for [94]. Surgery will always have a role in the management of patients — be that emergency life/limb saving surgery, oncological operations or symptomatic cures — and it is important we can continue this function unabated.

We are yet to see the long-term sequalae of the pandemic — both the direct sequalae (such as long COVID and post-COVID lung disease) and the indirect sequalae (including later diagnosis and treatment, physical and psychological deterioration). These issues also apply to the staff who have been at higher risk throughout the pandemic. Healthcare workers are the backbone of any healthcare delivery, and it is imperative we address the efflux of personnel and combat the high levels of burnout amongst staff.

Vaccination programmes are progressing, but the disparity in access makes the potential for more lethal or transmissible variants a real possibility — threatening to throw us back to March 2020. It will be interesting to see the outcomes of this.

The environments we work and live in should be intelligently designed to reduce the transmission risks and improve standard of living, especially pertinent when the disparity between survival between the most and lease affluent is so stark. Hospital design going forward should consider the implications of highly transmissible diseases and mechanisms for reducing nosocomial transmission.

## Conclusions

The COVID-19 pandemic has put surgical disciplines under severe strain, but surgical teams have been quick to rise to the challenge. They have responded to this new threat by developing consensus practical guidelines, establishing research priorities, examining safety precautions and understanding outcomes of different surgery during the pandemic. Overall, it is clear that surgery in patients with a peri-operative SARS-CoV-2 infection is associated with pulmonary complications and mortality in a very large number. Therefore, surgery should be avoided in these patients, and preference should be given to conservative management if it has a reasonable chance of succeeding.

However, non-emergency surgery allows for adequate precautions. These patients should undergo screening for symptoms, preoperative testing using RT-PCR within 72 h of surgery, preoperative self-isolation after the testing (and for 2 weeks in high-risk groups), treatment in COVID-free or minimal facilities and postoperative self-isolation if they belong to high-risk groups such as age > 75 years, ASA > 2 and immunocompromised individuals.

Trainers should be given more time and resources for focussed surgical skills training using simulation and videos, and training programmes should consider developing the role of dedicated technical skills’ mentors for each trainee.
